# Full-Genome Sequences of Seven Fatal Enterovirus 71 Strains Isolated in Shenzhen, China, in 2014

**DOI:** 10.1128/genomeA.00316-16

**Published:** 2016-04-28

**Authors:** Long Chen, Ya-Qing He, Jun Meng, Ling-Hong Xiong, Chao Wang, Xiang-Jie Yao, Hai-Long Zhang, Ren-Li Zhang, Hong Yang

**Affiliations:** a Major Infectious Disease Control Key Laboratory, The Department of Microbiology, Shenzhen Center for Disease Control and Prevention, Shenzhen, People’s Republic of China; b Shenzhen Public Service Platform of Pathogenic Microorganisms Repository, The Department of Microbiology, Shenzhen Center for Disease Control and Prevention, Shenzhen, People’s Republic of China

## Abstract

The whole-genome sequences of seven fatal enterovirus 71 (EV71) strains, isolated in southern China, in 2014, were determined. The complete genome sequences of these strains displayed close relationships to native EV71 strains and showed 94.2% to 99.8% identity to each other. All of these strains were assigned to subgenotype C4a based on phylogenetic analysis of the VP1 gene.

## GENOME ANNOUNCEMENT

Enterovirus 71 (EV71), a member of the enterovirus A species of the family *Picornaviridae*, is the most common pathogen of hand, foot, and mouth disease (HFMD) in children and infants (1, 2). Enteroviruses are a group of naked positive single-stranded RNA viruses, and their genome comprises a 5′ untranslated region (UTR), structural polypeptide P1, nonstructural polypeptides P2 and P3, and a 3′ untranslated region (3). Based on phylogenetic analysis of the VP1 genes, worldwide EV71 strains were classified into 3 main genogroups (A, B, and C) and 12 subgenotypes (A, B0 to B5, and C1 to C5) (4–6). Epidemic waves of EV71 infections have swept through countries in the Asia-Pacific region since 1997 (7, 8). Although the globally emerging pathogen coxsackievirus A6 (CV-A6) has tended to predominate over EV71 as the etiologic agent of HFMD since 2013, EV71 is still responsible for severe HFMD and even death (9–11).

From March 2014 to September 2014, the Shenzhen Center for Disease Control and Prevention (CDC) received death reports of eight children with severe HFMD infections. Fecal specimens or anal swabs from these cases were archived at the Department of Microbiology, Shenzhen CDC. Seven of 8 specimens (87.5%) were detected as positive for EV71 by real-time reverse transcription-PCR (RT-PCR), and the other specimen (1/8 [12.5%]) was detected as positive for CV-A16 (12). Next, these strains were isolated by culturing clinical samples in rhabdomyosarcoma (RD) cell lines. Detailed epidemiological data for seven fatal EV71 strains are listed in Table 1.

**TABLE 1:** Epidemiological data for seven fatal EV71 strains from this study

Isolate	Gender	Age (yr)	Clinical manifestation	Accession no.
EV71/SZ04/CHN/2014	Female	1.6	Fever; rash; vesicles on hands and buttocks; pharyngalgia; lethargy; polypnea; rapid heart rate; muscle twitching; abnormal eye movements; and aseptic encephalitis	KT428644
EV71/SZ07/CHN/2014	Female	1.2	Fever; rash; polypnea; cold limbs; shock; and bronchopneumonia	KT428645
EV71/SZ12/CHN/2014	Male	2.5	Fever; rash; vesicles on hands, foot, and mouth; polypnea; and aseptic encephalitis	KT428646
EV71/SZ25/CHN/2014	Male	2.6	Fever; rash; vesicles on hands, foot, and mouth; polypnea; rapid heart rate; and muscle twitching	KT428647
EV71/SZ42/CHN/2014	Male	4.3	Fever; vesicles on hands, foot, mouth, knees, and buttocks	KT428648
EV71/SZ50/CHN/2014	Male	3.0	Fever; rash; vesicles on hands, foot, and buttocks; muscle twitching	KT428649
EV71/SZ88/CHN/2014	Male	1.9	Fever; rash; vesicles on hands, foot, mouth, elbow, knees, and buttocks; cough; lethargy; polypnea; rapid heart rate; muscle twitching; and aseptic encephalitis	KT428650

A pair of universal primers, EVA-F30 (5′-TTAAAACAGCCTGTGGGTTGTACCCACCCA-3′) and EVA-R36 (5′-GCTATTCTGGTTATAACAAATTTACCCCCACCAGTC-3′), were used to amplify full-length genomes of these strains by a one-step RT-PCR method, as described previously (12). Amplified DNA products were sequenced by TaKaRa (Japan) using a primer-walking method. Contigs were assembled using sequencer version 4.9. The raw genome sequences were examined using BioEdit version 7.2.5 before submission to GenBank. Molecular phylogeny was investigated using the program MEGA 6.06 (13).

The complete genome sequences of seven EV71 strains were composed of 7,405 nucleotides (nt), excluding the poly(A) tail. The 5′-UTR was found to be 742 nt, followed by an open reading frame (ORF) encoding the structural protein P1 (2,586 nt), the nonstructural proteins P2 (1,734 nt) and P3 (2,259 nt), and the 3′-UTR (81 nt). The contents of A, C, G, and U of the seven EV71 genome sequences were 27.05 to 27.27%, 23.97 to 24.19%, 23.70 to 23.92%, and 24.83 to 25.02%, respectively, with G+C contents of 47.67 to 48.11%. The full-length genome sequences of these strains displayed close relationships to native EV71 strains and showed 94.2 to 99.8% identity to each other. All of these strains were assigned to subgenotype C4a based on phylogenetic analysis of the VP1 gene.

To date, a vaccine against EV71 has not been commercially introduced (14, 15), but family- and kindergarten-based early intervention programs and timely treatment may reduce the incidence of severe and fatal cases.

### Nucleotide sequence accession numbers.

The full-length genome sequences of seven fatal EV71 isolates have been deposited in GenBank under the accession numbers listed in Table 1.
